# Incidence of Complications in Chest Wall Masculinization for the Obese Female-to-Male Transgender Population: A Case Series

**DOI:** 10.29252/wjps.10.2.14

**Published:** 2021-05

**Authors:** Idanis M. Perez-Alvarez, Elizabeth G. Zolper, Jonathan Schwitzer, Kenneth L. Fan, Gabriel A. Del Corral

**Affiliations:** 1 Georgetown University School of Medicine; Washington, District of Columbia, USA.; 2 Department of Plastic and Reconstructive Surgery, MedStar Georgetown University Hospital; Washington, District of Columbia, USA.; 3 Plastic and Reconstructive Surgery, MedStar Franklin Square Medical Center; Baltimore, Maryland.

**Keywords:** Chest masculinization, Transgender, Gender affirmation, Female-to-male, Subcutaneous mastectomy

## Abstract

**BACKGROUND:**

Chest masculinization is aimed at aligning physical appearance of female-to-male (FtM) transgender patients to their identifying gender. Despite limited evidence, obese FtM patients have historically been denied this procedure due to concerns of complications. We reviewed chest masculinization in the high body mass index (BMI) population to analyze the outcomes.

**METHODS:**

A Medstar system single surgeon retrospective case review was performed of all FtM patients who underwent chest masculinization from Jan 2018 to Dec 2019 with a BMI greater than 30 kg/m^2^. Primary outcomes were mastectomy-site complications.

**RESULTS:**

Twenty-seven obese FtM patients who underwent bilateral chest masculinization were identified. Mean BMI was 39.2 kg/m^2 ^(SD 5.2). Preoperatively, the majority of patients had a cup size of D or larger (77.3%) and grade 3 ptosis (80.0%). Overall rate of complications was 31.5% at median follow-up of 2.1 months. Individual complications included: partial nipple graft loss 18.5%, total nipple graft loss 5.6%, seroma 3.7%, hematoma 3.7%, infection 2.9%. No complications necessitated return to the operating room. However, the majority of patients (77.8%) were completely satisfied with their aesthetic outcome.

**CONCLUSION:**

Mastectomy can be safely performed for chest masculinization in obese FtM patients. The rate of acute complications is comparable to that of non-obese patients despite a mean BMI near 40 kg/m^2^ in this case series. A safe procedure with high satisfaction, obese FtM patients should not be excluded from the increased quality of life and dysphoria reduction chest wall masculinization offers.

## INTRODUCTION

Acceptance of gender dysphoria as a clinical diagnosis and widespread societal exposure to transgender individuals has led to an increased demand for gender-confirming surgical intervention^1^^,^^2^. Chest wall masculinization is the most common procedure performed to facilitate the transition from female-to-male^3^. While not an essential component to ameliorating gender dysphoria, this gender-confirming procedure can offer increased quality of life^4^. 

Clinically, “top surgery” as it is commonly referred to, is aimed at aligning physical appearance of a phenotypic female to their identifying male gender through a technique similar to that of mastectomy. However, it is important to recognize significant differences among the procedures. Extensive excision of breast tissue including the axillary tail of Spence is performed to ensure that all hormonal glands are eliminated from the body and to eliminate the need for future breast screening in these patients. Additionally, advanced techniques for nipple-areolar complex grafting have been developed to accentuate the male phenotype. Chest contouring and reduction are other surgical methods utilized to assimilate the male appearance. 

Evidence of outcomes for chest masculinization is limited. Generally, it is accepted among plastic surgeons that patient characteristics that predispose to surgical complications are equally applicable to this population. Breast size, degree of ptosis, and skin elasticity are largely predictive of the amount of skin resection required and aesthetic outcome of the procedure. Increased body mass index (BMI) is thought to be associated with poor surgical outcomes. Despite lack of supporting evidence, those with a BMI above 30 kg/m^2^ have historically been denied from receiving this procedure.

Herein, we present a case series of chest wall masculinization in twenty-seven obese transgender patients and present post-operative outcomes for this cohort.

## METHODS

***Retrospective review***

Following Institutional Review Board approval (IRB 2018-173), a single-center retrospective review was performed of all female to male transgender (FtM) patients who underwent mastectomy for chest wall masculinization at Medstar Franklin Square Hospital, Washington, USA from Jan 2018 to Dec 2019. All patients satisfied the following inclusion criteria: 1) chest masculinization performed by the senior author (GDC); 2) BMI greater than 30 kg/m^2^, and 3) documented follow up. 

Data collected included patient demographics (age, sex, and medical comorbidities), operative details, postoperative complications, and aesthetic outcomes. The primary outcome was mastectomy-site complications which included nipple graft loss, seroma, hematoma, infection, and delayed healing.

***Operative technique***

To start the chest masculinization procedure, an ellipse of skin and subcutaneous tissue, including the nipple-areolar complex (NAC), is excised. Dissection is carried down to the level of the pectoralis major fascia. Each nipple is circumscribed with an areola marker, with diameters ranging from 18-22 mm and excised as a full-thickness graft. The free nipple grafts (FNGs) are defatted for maximal graft viability. The nipples should be partially thinned, removing only the subcutaneous tissue while preserving the dermis. This maneuver will prevent postoperative nipple contraction and hypopigmentation. The patient is then moved upright to facilitate positioning of the FNGs. Using the areolar marker, the desired location for the FNG is marked and de-epithelialized. The FNGs are then sutured in place. 

Then an incision is made in the inframammary fold (IMF) to allow for removal of the breast specimen. The thickness of the superior mastectomy flap is now matched to the inferior mastectomy flap to prevent postoperative chest wall concavity. In the obese patient, it is advisable to leave the superior mastectomy flap a few millimeters thicker, as this will result in a more natural contour of the chest consistent with a patient with more chest subcutaneous tissue. Blake drains are placed in the pre-pectoral space and the skin is approximated with staples. After bilateral excision of breast tissue, the patient is seated in the upright position in order to assess symmetry of the chest mounds. Suction assisted lipectomy is performed over the deltopectoral triangle and the lateral chest wall to minimize any dog-ears. The IMF is then closed with the closed-suction drains secured laterally, dressings are applied, followed by elastic bandages to provide compression.

***Statistical analysis***

Continuous variables were described as mean and standard deviation or median and interquartile ranges as determined by the Shapiro-Wilk test of normality. Two sample t-test and Wilcoxon Rank Sum test were used to compare continuous variables between groups as appropriate. Categorical variables were described by frequencies and percentages. Fisher exact tests were used to compare proportions of categorical variables. Statistical analysis was performed using STATA ver.16 (StataCorp, College Station, Texas) with significance defined as *P*<0.05.

***High BMI Incision Algorithm***

Subsequently, an algorithm for incisional approach in the obese FtM population was developed. The potential operative techniques demonstrated in previous literature, as well as studies of cosmetic breast surgery in high BMI patients, were compared with the senior author’s (GDC) experience with these patients ^3^^,^^5^^-^^10^.

## RESULTS

***Demographics***

We identified twenty-seven FtM patients with BMI greater than 30 kg/m^2^ who underwent bilateral mastectomies for chest wall masculinization (Table 1). Mean age at time of chest wall masculinization was 26 yr (SD 5) with mean BMI of 39.2 kg/m^2 ^(SD 5.2). Common comorbidities were: major depressive or generalized anxiety disorder 70.4% and diabetes 14.8%. Four patients were known to be active smokers within four weeks of their operation (Table 2). The majority of patients had a cup size of D or larger (n=17/22, 77.3%) and grade 3 ptosis (n=20/25, 80.0%).

***Operative details***

 Fifty-four mastectomies were performed for chest wall masculinization (Table 2). All cases were bilateral. The mean operative time was 108 min (SD 20). Median total weight of mastectomy specimens was 2070 grams (IQR 1593-2574). There were no intraoperative complications. Median time to drain removal was 8.5 days (IQR 7-13). Median follow-up was 2.1 months (IQR 1.0-4.8). 

**Table 1:** Patient demographics and comorbidities

**Demographics**	
Total number of patients	27 (100)
Age in years; mean ± SD	26 ± 5
BMI in kg/m^2^; mean ± SD	39.2 ± 5.2
**Comorbidities**	
Diabetes mellitus	4 (14.8)
Smoking history	
Never	18 (66.7)
Prior	5 (18.5)
Active^a^	4 (14.8)
Obesity	27 (100)
Hypertension	3 (11.1)
MDD or GAD	19 (70.4)

*SD—standard deviation, BMI—body mass index, MDD—major depressive disorder, GAD—generalized anxiety disorder.* *All numbers are presented as n (%) unless otherwise noted.* ^a^ *Active smoker defined as within four weeks of operation.*

***Postoperative complications***

Complications occurred for seventeen mastectomy sites (31.5%) (Table 3). The most common complication was partial FNG loss (n=10/54, 18.5%). Total FNG loss occurred in three mastectomy sites (5.6%). Complications required in-office procedures for four mastectomy sites (7.4%) in four patients: 1 secondary closure after dehiscence, 1 hematoma evacuation, and 2 seroma evacuation. No complications necessitated return to the operating room.

Two patients known to be active smokers within four weeks of their operation experienced complications. First, one patient experienced seroma and infection both affecting the same mastectomy site. Additionally, this patient was noted to have superficial epidermolysis of their nipples. The second patient developed unilateral partial FNG loss. Both of these cases, the pigmentation in the nipples returned at 12 and 16 months, respectively. However, smoking history was not statistically significant for incidence of overall (*P*=0.527) or FNG specific complications (*P*=0.325) (Table 4).

**Table 2:** Perioperative characteristics

**Preoperative characteristics**	
Breast Size^a^	
B cup	1 (4.5)
C cup	4 (18.2)
D cup	10 (45.4)
DD cup	6 (27.3)
DDD cup	1 (4.5)
Degree of Ptosis^b^	
Grade 2	5 (20.0)
Grade 3	20 (80.0)
**Operative characteristics**	
Total number of mastectomy sites	54 (100)
Operative time in min; mean ± SD	108 ± 20
Total specimen weight in grams; median (IQR)	2070 (1593-2574)
Time to drain removal in days; median (IQR)	8.5 (7-13)
Follow up in months; median (IQR)	2.1 (1.0-4.8)

*SD—standard deviation, IQR—interquartile range.* *All numbers are presented as n (%) unless otherwise noted.* *Frequencies for* ^a^ *breast size and* ^b^ *ptosis are out of 22 and 25 patients, respectively, due to incomplete data*

***Aesthetic outcomes***

The majority of patients were satisfied with the aesthetic outcome of the procedure. 77.8% of patients were found to be content after their chest masculinization, measured by chart review for either reported complaints about aesthetics or incidence of secondary revisional procedures. Poor aesthetic outcomes were documented by the senior author and were expressed regarding thirteen mastectomy sites (24.1%) (Table 3). Poor scarring and contour abnormalities occurred in 18.5% and 11.1% of mastectomy sites, respectively. Cosmetic revision was performed for three mastectomy sites (5.6%) in two patients. All revisions were minor and performed in office. No revisions necessitated return to the operating room.

**Table 3:** Mastectomy site complications and aesthetic outcomes

**Postoperative complications**	
Any complication	17 (31.5)
Partial FNG loss	10 (18.5)
Total FNG loss	3 (5.6%)
Seroma	2 (3.7)
Hematoma	2 (3.7)
Infection	1 (2.9)
Delayed healing	1 (1.9)
Procedure required	
In office	4 (7.4)
Return to operating room	0 (0)
**Aesthetic outcomes**	
Any aesthetic concern	13 (24.1)
Poor scarring	10 (18.5)
Contour abnormality	6 (11.1)
Cosmetic revision	
In office	3 (5.6)
Return to operating room	0 (0)
Aesthetic satisfaction per number of patients	21 (77.8)

***Bivariate analysis***

The relationship of demographics, comorbidities, and key perioperative characteristics on outcomes (any complication, any FNG loss, aesthetic satisfaction) were analyzed per patient. The only factor reaching significance was age at time of operation which was significant for incidence of any complication (*P*=0.046, Table 4). Body mass index was not significant for any outcome of interest; however, patients who developed complications, FNG loss, or were unsatisfied with their aesthetic outcome tended to have lower BMI on average (Table 4). Total mastectomy specimen weight also was similar between groups for all three outcomes with patients who developed complications, FNG loss, or were unsatisfied with their aesthetic outcome having a lower median specimen weight (Table 4).

**Table 4:** Demographics, comorbidities, and perioperative characteristics by patient outcomes

**Variable**	**Any Complication**			**Any FNG Loss**			**Aesthetic Satisfaction**		
	**Yes** ***N=12***	**No** ***N=15***	***P***	**Yes** ***N=8***	**No** ***N=19***	***P***	**Yes** ***N=21***	**No** ***N=6***	***P***
**Demographics**									
Age in years; mean ± SD	28 ± 5	24 ± 4	0.046*	27 ± 5	25 ± 5	0.265	26 ± 5	24 ± 4	0.394
BMI in kg/m^2^; mean ± SD	38.5 ± 5.9	39.7 ± 4.6	0.564	38.9 ± 7.2	39.3 ± 4.3	0.884	39.7 ± 5.1	37.4 ± 5.4	0.350
**Comorbidities**									
Diabetes mellitus	2 (16.7)	2 (13.3)	1.000	1 (12.5)	3 (15.8)	1.000	4 (19.0)	0 (0)	0.545
Smoking history			0.527			0.325			0.319
Never	9 (75.0)	9 (60.0)		7 (87.5)	11 (57.9)		15 (71.4)	3 (50.0)	
Prior	1 (8.3)	4 (26.7)		0 (0)	5 (26.3)		4 (19.0)	1 (16.7)	
Active	2 (16.7)	2 (13.3)		1 (12.5)	3 (15.8)		2 (9.5)	2 (33.3)	
Hypertension	1 (8.3)	2 (13.3)	1.000	0 (0)	3 (15.8)	0.532	3 (14.3)	0 (0)	1.000
MDD or GAD	8 (66.7)	11 (73.3)	1.000	6 (75.0)	13 (68.4)	1.000	13 (61.9)	6 (100)	0.136
**Perioperative characteristics**									
Degree of Ptosis^a^			1.000			1.000			1.000
Grade 2	2 (16.7)	3 (23.1)		1 (12.5)	4 (23.5)		4 (20.0)	1 (20.0)	
Grade 3	10 (83.3)	10 (76.9)		7 (87.5)	13 (76.5)		16 (80.0)	4 (80.0)	
Operative time in min; mean ± SD	106 ± 19	110 ± 22	0.599	103 ± 19	110 ± 21	0.436	105 ± 19	120 ± 23	0.108
Total specimen weight in grams; median (IQR)	1919 (1434-2239)	2308 (1675-2877)	0.399	1811 (1434-2239)	2117 (1675-2701)	0.482	2070 (1675-2701)	1896 (1275-2126)	0.408

*Statistically significant (P<0.05). SD—standard deviation, BMI—body mass index, *MDD—major depressive disorder, GAD—generalized anxiety disorder, IQR—interquartile range.* *All numbers are presented as n (%) unless otherwise noted.* ^a^ *Frequencies for* *degree of ptosis are out of 12, 13, 8, 17, 20 and 5 patients from left to right, respectively, due to incomplete data.*

## DISCUSSION

Our experience demonstrates that chest wall masculinization can be safely performed in FtM patients with a BMI above 30 kg/m^2^. The goals of chest masculinization are aesthetic contouring of the chest wall, proper reduction and position of the nipple, and minimization of chest wall scars^11^. Often, obese individuals are denied this procedure due to concerns of compromise to one of these four goals. However, rates of complications and reoperation in the obese population are acceptable and should not preclude them from being offered chest masculinization.

 Previous studies estimate overall complication rates associated with chest masculinization to range from 11% to 50% ^3^^,^^5^^,^^6^^,^^10^^,^^12^. Overall, 346 mastectomies were performed for FtM chest masculinization with an overall complication rate of 11.8% of mastectomy sites ^6^. Our overall rate of complications was notably higher at 31.5%. However, all complications in our series were able to be managed with conservative, in office measures, while previous reports a 10.4% incidence of major, operative complications. Similarly, we also report a lower rate of cosmetic revision at 5.6% all of performed in office compared to a 9% revision rate in the prior study. Thus, we believe this incidence of minor complications is acceptable given the immense significance of chest masculinization to patients. 

Uniquely, our study chose to focus on outcomes in the obese population for which there is paucity of literature to date. Wolter et al. had a mean BMI was 23.93 kg/m^2 ^(range: 17.1 -39.5 kg/m^2^) distinct from our study which had a mean of 39.5 kg/m^2 ^(range of 30.7-50 kg/m^2^). A prior study that stratified rates of complications by BMI demonstrated that rates of major complications were increased in those with normal weights rather than those who were obese or extremely obese (55% vs. 27% vs. 0%, respectively) ^5^. Therefore, it is important to recognize the lack of validity of previous literature as a means to make conclusions of risk profiles for obese FtM patients. 

**Fig. 1:**
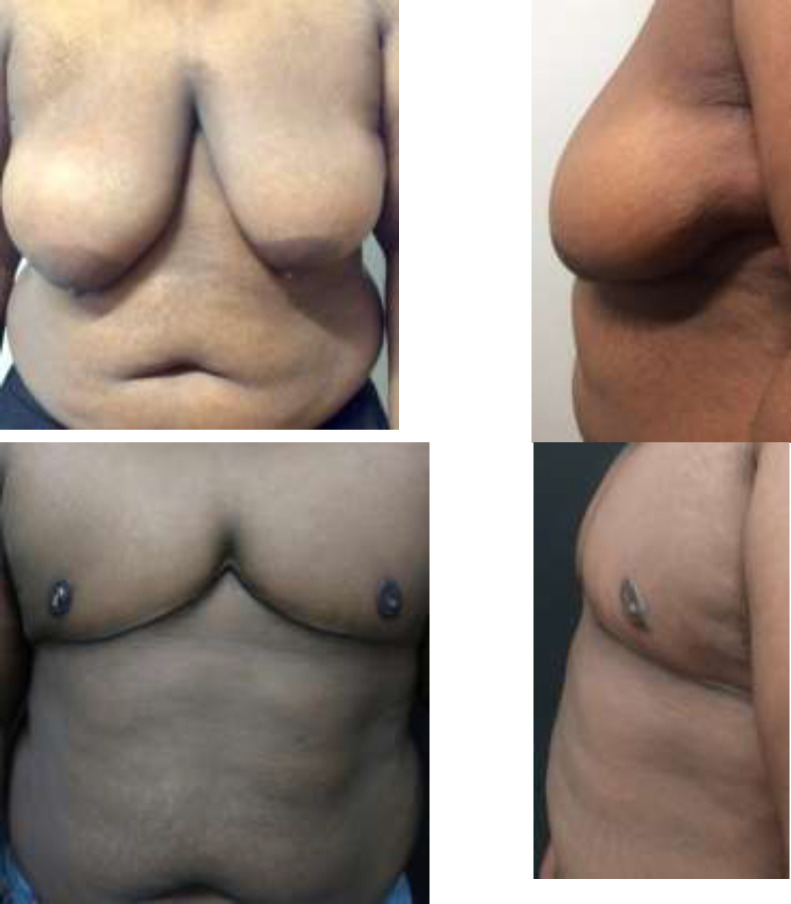
22-year-old FtM patient with a BMI of 40.62 kg/m^2^ and D cup breasts with grade 3 ptosis. Anterior (A) and left lateral (B) preoperative photos. Weight of right and left mastectomy specimens were 1317g and 1048g, respectively. An inframammary incision with lateral extension was utilized. Anterior (C) and left lateral (D) at 11 wk post operation. There were no complications

Partial NAC necrosis was the highest contributor to complications, occurring in ten mastectomy sites (18.5%). In prior studies, rates of partial NAC necrosis vary from 0.9%-17.9% ^5^^,^^10^^,^^12^^,^^13^. We defined partial NAC necrosis to include any skin changes greater than 5mm. Partial NAC necrosis rate was likely increased in our high BMI population due to low threshold for diagnosis of partial NAC. Another possible explanation includes more extensive dissection and excision of skin needed to achieve a good contour, and post-operative compression which likely decreases local perfusion thus reducing FNG take^14^. Figure 1 demonstrates preoperative and postoperative images of a patient who experienced NAC necrosis managed successfully with Santyl and resolved without need for reoperation.

**Fig. 2:**
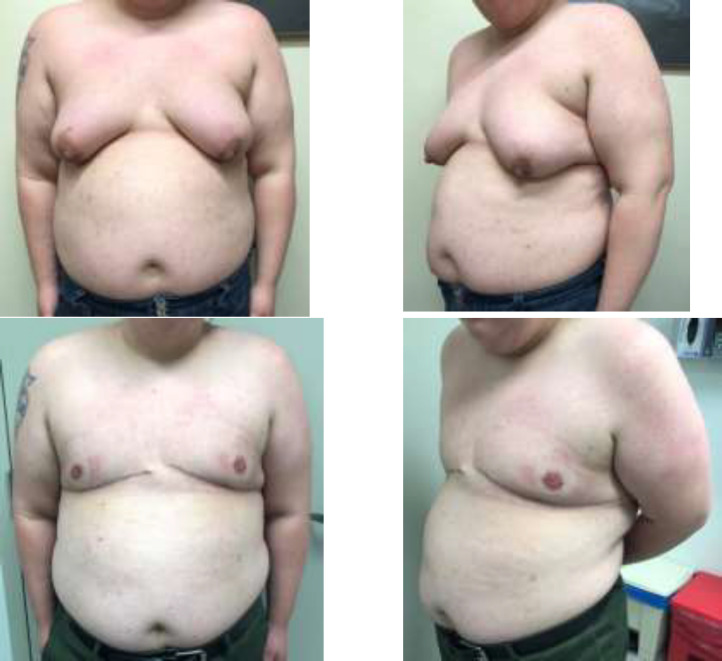
27-year-old FtM patient with a BMI of 40.31 kg/m^2^ and C cup breasts with grade 3 ptosis. Anterior (A) and left lateral (B) preoperative photos. Weight of right and left mastectomy specimens were 197g and 675g, respectively. Anterior (C) and left lateral (D) at six weeks post operation which utilized an inframammary incision with lateral extension. There was loss of his left central areolar nipple graft managed conservatively with application of Santyl

Understandably, the greatest concern in chest masculinization in the obese population is the known association between larger breast size and chest scarring. This observation made early in “Amsterdam experience” studies^11^. Many other studies regarding FtM top procedures have further investigated this relationship^3^^,^^6^^,^^10^^,^^13^^,^^15^^,^^16^. Our patient population varied in breast sizes but predominantly skewed towards higher volumes, with 77% D cup or larger and 80% grade 3 ptosis. In regards to our results, five patients (18%) experienced poor scarring. This compares to another study ^17^, in which 8% of patients who underwent FNG required scar revision, a majority of whom had Fischer grade 3 breasts. Increased chest wall scarring was preferred over excess skin, which would lead to other contour abnormalities ^3^. The majority of patients did not experience any major complications; median total mastectomy specimen weight was higher in patients with good outcomes in our series but did not reach significance. Figure 2 demonstrates pre and post-operative views of an obese, large breasted patient with minimal scarring and ideal chest contouring representative of our patient results. 

***Surgical Approach***

Surgical technique is critical to optimizing aesthetic results in FtM patients, especially those with larger breast volumes. Robust literature exists proposing preferred surgical approaches to chest masculinization depending on breast size. In the 1990s, Hage and Bloem published their “Amsterdam experience” in which they outlined three approaches to chest masculinization guided by preoperative breast size and mastoptosis^11^. For patients with significant ptosis or large breast volumes, they performed horizontal extension to the NAC and fusiform excision with FNG. 

Subsequently, an algorithm was proposed for choosing the most suitable approach for mastectomy, depending on the breast size and envelope, NAC form and position, and skin elasticity ^3^. Skin excess and skin elasticity were the principal factors influencing technique. Patient outcomes were analyzed based on four surgical techniques applied to a patient population based on cup size, ptosis, and skin elasticity. Patients with C-D cup size, grade 1 ptosis, and moderate to poor elasticity underwent inferior pedicled mastectomy. Whereas, mastectomy with free NAC graft was performed in those with >D cup, grade 3 ptosis, and poor elasticity. This last group is most similar to our study cohort of patients (Table 2).

Outcomes of surgical techniques were analyzed including no skin resection, periareolar skin resection, and inframammary skin resection with either pedicled nipple or FNG ^10^. In their study, nipple/areolar correction was significantly associated with periareolar resection when compared to FNG and scar revision was performed more often after pedicled nipples than FNG. Despite non-significance, chest contouring correction was performed more frequently after periareolar excision. Similarly, transverse IMF incision was not only associated with significantly reduced rated of revision (8% IMF vs 25% periareolar), but was also associated with decreased rates of complications (22.7% vs 35%) ^5^.

**Fig. 3:**
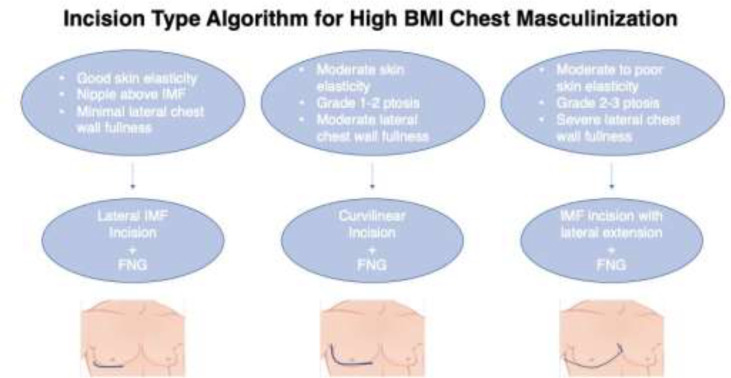
Algorithm for three subcutaneous mastectomy incision techniques in female-to-male chest masculinization with high BMI. *BMI—body mass index, IMF—inframammary fold, FNG—free nipple graft*

Based on our experience with twenty-seven obese FtM patients, we present an algorithm for incision technique in this population (Figure 3). These recommendations underscore the importance of nipple-grafting in obese patients corroborated by previous literature of chest masculinization in large breasted FtM patients^3^^,^^10^. However, we propose utilization of inferior breast incisions, which differs from previously described radial or periareolar approaches. An inferior breast incision is preferred due to increased access for excision of breast tissue which is critical to achieving an aesthetic contour in the obese population. While this approach may leave a noticeable scar beneath the new male breast, it is preferred to avoid potential complications associated with periareolar excisions. Therefore, in obese patients with large breasts, the favorable approach is an inferior incision with FNG.

Additionally, we preferred the addition of a lateral extension of the inferior incision particularly in patients with poor skin elasticity, and Grade 2-3 ptosis. This technique was frequently utilized in this study given that the majority of our FtM patients fit into this categorization. A combination of surgeon experience and literature regarding breast reshaping in the post-bariatric surgery population motivated the adoption of this approach. Bariatric literature commonly references lateral extension of the Wise pattern for augmentation or mastopexy to correct lateral thoracic skin redundancy or “side rolls” ^7^^–^^9^. With a similar goal of improving chest contouring in our patients, lateral extension of the inferior incision allows for resection of excess lateral chest wall skin to achieve further improved aesthetic outcomes of chest wall masculinization in obese FtM patients.

***Quality of life***

Those identifying as transgender not only have to navigate a world in a discordant-gendered body, many of them suffer from a myriad of comorbid conditions secondary to their indisposition. In our study, prevalence of major depressive or generalized anxiety disorder was 70.4%. Donato et al.^5^ had a combined rated of 53.1% of patients with mental health history, either active or resolved. Across the board, mental health quality of life is less in transgender FtM than cis-gender females^18^. Chest masculinization is one of how plastic surgeons can help alleviate many of the insecurities of transgender individuals. 

Increased quality of life is a critical component of providing this procedure to those that assimilate to a male gender despite a female phenotype. While chest masculinization may not be desired or indicated for all FtM, improvements in psychological function have been shown to significantly improve after gender confirmation surgery^19^^–^^21^. Patients were followed longitudinally through phases of puberty suppression, cross-hormone introduction, and surgery ^22^. After gender confirmation surgery, individuals demonstrated alleviation of gender dysphoria, increased psychological functioning, and overall wellbeing^22^. 

Use of validated patient-reported outcome measures (PROMs) have further demonstrated the impact of chest wall masculinization. Prior studies have utilized PROMs including Breast-Q, Body Uneasiness Test (BUT-A), and Short Form 36-Item Questionnaire version 2 (SF36v2) and confirmed significant improvements in satisfaction, psychological, and physical well-being after surgery^4^^,^^18^^,^^23^^–^^26^. Significantly, those with underlying mental conditions had the greatest improvements in Breast-Q and BUT-A survey scores^4^. 

Generally, transmasculine patients have larger breast volumes, greater amounts of excess skin, and ptosis^27^. High BMI patients tend to have larger breasts, making it more difficult to live as a man publicly and privately. Many of these individuals have resorted to breast binding to prevent visibility. However, this can increase skin elasticity, requiring excess skin excision during chest masculinization in the future^3^^,^^11^. As a large proportion of FtM patients are obese, early access to surgery in their gender affirmation transition can contribute to optimal cosmesis and satisfaction. 

 Despite concerns for poor cosmetic outcomes, postoperative complications are not increased in obese patients^5^^,^^6^^,^^16^. Furthermore, the slight predisposition to poor scarring or contour abnormality may not outweigh the psychological benefits. More recently, Black et al.^28^ studied social media posts of postoperative outcomes following chest wall masculinization. Comments from patients demonstrated a high level of satisfaction with surgical results despite receiving low aesthetic-quality ratings from board-certified plastic surgeons. This study highlights the psychological and functional impacts of surgery on gender dysphoria, of which quality of cosmetic outcome may not be the major driver of improved post-operative quality of life.

***Limitations***

Our study is limited by small cohort size. This is given the relatively recent transition to providing subcutaneous mastectomy for FtM transition at our institution and the relatively low proportion of patients with BMI greater than 30 kg/m^2^. However, the use of an exclusively obese population is also a strength due to its novelty. Furthermore, this study had a short follow-up period, which may not have elucidated rates of long-term surgical complications in this population. Regardless, acute complications were low and were managed effectively without reoperation for all patients. Moreover, lack of standardized analysis of patient satisfaction prevented conclusions of the psychological impact of chest wall masculinization on obese individuals. However, the study benefits from having a diverse cohort, varying in skin color and comorbidities, increasing external validity. Consequently, a single surgeon performed the same surgical technique on all patients, strengthening internal validity. Future studies with longer follow-up, a larger study group, and use of validated PROMs are warranted to understand the full impact of BMI on surgical complications or revision surgery following chest wall masculinization. 

## CONCLUSION

In an already discredited population, barriers to access to appropriate medical care should be heavily reduced for transgender individuals. Weight should not be a contraindication to chest masculinization surgery. Mastectomy can be safely performed for chest wall masculinization in obese FtM patients. While concerns for increased scarring and worse aesthetic outcomes are valid given the correlation between obesity and breast volume, the rate of acute complications is comparable to that of non-obese patients despite a mean BMI of nearly 40 kg/m^2^ in this case series. Furthermore, gender-affirming surgery confers significant benefits to satisfaction, psychological functioning, and wellbeing, regardless of quality of cosmesis. Overall, chest wall masculinization remains a safe procedure with high satisfaction in the obese population, thus, these individuals should not be excluded from the increased quality of life it offers. 

## FINANCIAL DISCLOSURE

There are no financial disclosures, commercial associations, or any other conditions posing a

## CONFLICT OF INTEREST

The authors declare that there is no conflict of interest.
